# Supplementary material for: Modeling the effects of contact-tracing apps on the spread of the coronavirus disease: Mechanisms, conditions, and efficiency
Source: PLoS One. 2021 Sep 1;16(9):e0256151. doi: 10.1371/journal.pone.0256151 (PMC8409648; doi:10.1371/journal.pone.0256151)
Supplement: S1 Appendix — (PDF) [file pone.0256151.s001.pdf]

Appendix

A. Computation of the duration of inactive time

The expected duration of being inactive conditional on becoming infected is derived by the following equation:

$$Duration = \frac{1}{N}\left(\sum_{a\in A}\sum_{s\in S, s\neq D} p_{as}d_{as} + \sum_{a\in A} p_{aD}d_{a\bar{D}}\right),$$

where p_{as} denotes the probability at which an infected person belonging to the age group a in all age groups A recovers at the stage s in all stages S except for death, d_{as} denotes the duration of their inactive time, and N denotes the total population. Similarly, p_{aD} and $d_{a\bar{D}}$ denote the probability and the duration of inactive time, respectively, for those who ultimately die. p_{as} is computed using the figures in Table 3. As for d_{as}, the infected people become inactive when they are hospitalized or become severely ill: If they are confirmed positive, they are hospitalized, and all their contact networks become inactive until they recover. Even if the infected people are unable to get tested, they initiate self-quarantine and subsequently proceed to the severely ill stage. This self-quarantine has almost the same social effect as hospitalization—it deactivates all contacts outside the home or nursing home (in case of the elderly) until recovery—except that it decreases community contact by 90%. The inactive period ends when the diagnosed person recovers. To summarize, d_{as} is derived by the following equation:

$$d_{as} = \sum_{0<i\leq d_M} p_{asM_i}\left((d_M - i) + d_{asS}\right) + \left(1 - \sum_{0<i\leq d_M} p_{asM_i}\right) d_{asS},$$

where p_{asM_i} denotes the probability with which an infected person belonging to the age group a and recovering at the stage s gets tested on the i'th day being in the moderate state, d_M denotes the expected duration of exhibiting the moderate state symptoms common to everyone (set to 8), and d_{asS} denotes the expected duration from becoming severely ill until recovery. Assuming that daily testing is conducted for randomly selected people, the day when they get tested in the moderate state follows the exponential distribution with the mean value equal to the inverse of the test probability.

If the infected people die, the duration of their inactive time should be treated differently from those in the other cases. If they die from the illness, the social cost mainly lies in the loss of their lifetime activities that would have been realized if alive rather than the loss arising from hospitalization or self-quarantine. Based on this assumption, the duration of inactive time of those who die in each age group a, $d_{a\bar{D}}$, is expressed as follows:

$$d_{a\bar{D}} = d_{aD} + \max\{L - a, 0\},$$

where d_{aD} denotes the duration of their inactive time until death, and L denotes the average lifespan (set to 85).

The expected durations averaged over age groups derived by this method are 61.0 days, 61.8 days, and 62.2 days if daily tests are conducted for 30%, 50%, and 70% of symptomatic people, respectively.

B. The impact of heterogeneity

This section discusses how agent-based models are different from analytical epidemiological models, namely, susceptible-infected-removed (SIR) models. [1, 2] In many analytical models, the number of newly infected people is determined by the product of the number of the infected and the number of the susceptible. The number of the severely ill and of the recovered are determined as a certain proportion of the infected. Thus, the overall mechanism of the transmission of virus in analytical models is equivalent to that in agent-based models, as presented in this paper. An obvious difference is that agent-based models take a bottom-up approach, meaning that the smallest unit in their structure is an individual, which enables analyses on inter-relationships among people. Fundamentally, this can be interpreted as high-dimensional heterogeneity. That is, in agent-based models, each individual is characterized by a variety of attributes and contacts. In other words, an agent-based model without this diversity is substantially the same as analytical epidemiological models.

Based on this view, cases are compared under scenarios with different degrees of heterogeneity (Table 1). In the baseline scenario, attributes of individuals, such as residence, age, industry, and job, are varied. The layers where they form contact networks are distinguished because the networks are constructed in accordance with their attributes. The assumption of super-spreading environments is also considered. In the scenario labeled "Age_Layer_SS Het.", residence is omitted from the set of attributes, so contact networks in all layers link people in a society. Similarly, the other scenarios partially omit the attributes in the baseline environment. In each scenario, parameters are set so that their population-averaged value is identical across scenarios.

Table 1. Scenarios with different degrees of heterogeneity.

Scenario	Super-spreader	Age	Job	Industry	Layers	Residence
Baseline (Pref_Layer_Age Het.)	Yes	Yes	Yes	Yes	Yes	Yes
Age_Layer_SS Het.	Yes	Yes	Yes	Yes	Yes	No
Age_SS Het.	Yes	Yes	No	No	No	No
SS Het.	Yes	No	No	No	No	No
Homogeneous	No	No	No	No	No	No

The results shown in Fig 1 suggest that a scenario with a high degree of heterogeneity tends to generate smaller estimates of cases than a scenario closer to perfect homogeneity. Specifically, the peak cases under the baseline in the main analysis

with full heterogeneity is less than 10% of those under the scenario with perfect homogeneity, which can be regarded as a simple SIR model realized in the present structure. In addition, the peak arrives more than two months later than in the scenario with homogeneity. Thus, it can be said that multi-dimensional heterogeneity strongly affects the estimation of cases and, accordingly, the simulated effects of various policy interventions on virus outbreak.

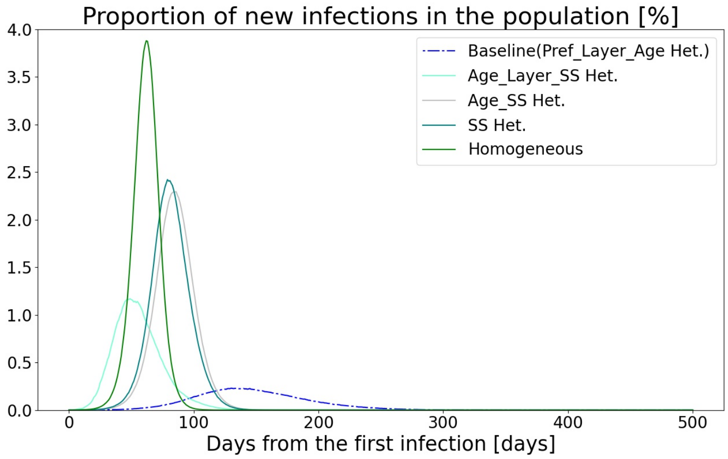

Fig 1. Cases and degree of heterogeneity.

References

1. Anderson RM, May RM. Population biology of infectious diseases: Part I. Nature. 1979;280(5721):361–367.

2. Kermack WO, McKendrick AG. A contribution to the mathematical theory of epidemics. Proceedings of the Royal Society of London Series A, Containing papers of a mathematical and physical character. 1927;115(772):700–721.
