# Supplementary material for: Modeling the effects of contact-tracing apps on the spread of the coronavirus disease: Mechanisms, conditions, and efficiency
Source: PLoS One. 2021 Sep 1;16(9):e0256151. doi: 10.1371/journal.pone.0256151 (PMC8409648; doi:10.1371/journal.pone.0256151)
Supplement: Attachment — Submitted filename: Reviewer2_second.pdf [file pone.0256151.s004.pdf]

Reviewer #2: The author has done an excellent job addressing my comments in the initial review. I suggest the following minor improvements:

*I deeply appreciate your insightful comments that have enhanced the quality of the paper. I am glad to hear that the first round of revision satisfied the concerns raised in the previous review. Following are the responses to the concerns raised in this second round of review.*

1.) Minor rewording and clarification of the abstract. Specifically, add a sentence just after the first sentence to briefly define the two responses to app alerts you are comparing (type-Q and type-T) and then edit the sentence beginning "First, with regard to contacts..." for English grammar and clarity.

***Response:*** *Thank you for your suggestion. I have revised the abstract so that the two types of responses are briefly defined and referred to.*

2.) Page 2: line 55 - 56 --The end of the sentence for point 1. of your main findings is at the beginning of point 2.

***Response:*** *Point 1 and 2 have been revised.*

3.) Page 4: line 133 -- The end of the sentence is missing.

***Response:*** *The codes are uploaded in protocols.io as suggested by the Editor. The sentence here has been revised to refer to the DOI of the uploaded file (http://dx.doi.org/10.17504/protocols.io.bwaepabe). The file is the same as the one submitted in response to the first-round review. Although the page in protocols.io is currently set as private, it is going to be published when the paper is published on the journal. The statement of no conflict of interest, originally inserted just after this sentence, has been removed because it was unnatural.*

4.) Page 4: line 135 - 136 -- Edit the first half of the first sentence under section 2.1 for English grammar and clarity.

***Response:*** *Thank you for the advice. The sentence has been revised.*

5.) Page 5: Section 2.2, line 165 - 166 -- For the sentence beginning "They might become infectious..." does the author mean asymptomatic but infectious? using the term "asymptomatic infectious" rather than simply "infectious" would help better define and differentiate between "infectious" and "symptomatic", since a symptomatic individual is also infectious.

***Response:*** *As pointed out, the term "infectious" in the sentence means asymptomatic but infectious. Therefore, it has been revised as suggested.*

6.) Page 5: Line 178 -- add the year after "June 1 to 7".

***Response:*** *The year 2020 has been added for clarity and correctness.*
