# Supplementary material for: Modeling the effects of contact-tracing apps on the spread of the coronavirus disease: Mechanisms, conditions, and efficiency
Source: PLoS One. 2021 Sep 1;16(9):e0256151. doi: 10.1371/journal.pone.0256151 (PMC8409648; doi:10.1371/journal.pone.0256151)
Supplement: Attachment — Submitted filename: Review of PONE-D-21-06447.docx [file pone.0256151.s002.docx]

*Study summary*

The purpose of this study is to determine the effectiveness of various scenarios and responses to app-based contact-tracing in terms of the cumulative proportion of the given population who must isolate/quarantine, with the understanding that reducing the overall number of individuals who must isolate/quarantine will result in a reduction in the negative economic impact of the COVID-19 pandemic within that population. In particular, the author uses agent-based modeling to compare populations responding to contact-tracing apps by testing for COVID-19 after being informed that they have been in proximity to an infected individual and quarantining only if their test result is positive (a type-T response) vs. populations responding to the apps by immediately quarantining without first testing to determine whether or not they have been infected (a type-Q response). Type-Q scenarios therefore result in quarantining individuals who do not actually have the virus, in addition to those who have the virus but do not yet know it because they are pre-symptomatic and have not yet been tested and/or received their test results. Type-T scenarios result in fewer individuals being quarantined as a result of proximity to an individual with a confirmed infection, but also result in asymptomatic or pre-symptomatic individuals who have been infected by the primary patient circulating throughout the community and potentially spreading infection to new individuals. This author’s model is based largely off of the model (called Covasim) published by Kerr et al. (2020) and available open-source via either GitHub or the Python Package Index. The author has extended Kerr et al.’s Covasim model in several ways by including such things as isolation of severely ill or hospitalized individuals (reducing their contact with others and thus the possibility of passing illness to another), adding “super-spreader” individuals to the model [see this reviewer’s comments on that later in this review], and adding contacts among service workers and their customers, among other things. All of these characteristics are randomly generated and interact to simulate spread of COVID-19 in a population according to the parameters assigned to them by the model. Conditions in addition to the type-T and type-Q responses that are varied for comparison in the simulations are percent of symptomatic individuals tested per day (for example, 30%, 50%, 90%), and the percent of smartphone users who download and use the contact-tracing app.

The results of this study can potentially be used to inform policy decisions related to COVID-19.

*How does this work differ from prior work? How does it add to the field?*

Most (all?) other published studies modeling contact tracing apps assume a type-Q response. This study adds to our understanding of the usefulness of the apps by adding the type-T response to the model.

Other agent-based models are quite complex, thus limiting the ability to determine the main factors that generate the effects seen in their simulations. This agent-based model is simplified and includes only the variables that the author deems necessary to achieve a reasonably realistic simulation in order to be able to tease out the mechanisms underlying simulation results [see this reviewer’s comments on the model assumptions below].

*Review*

Overall I was impressed by the premise of the study, and feel that the use of agent-based modeling to reveal the most important factors affecting population-based spread of COVID-19 infections has great potential. The author’s focus on contact-tracing apps in this effort is very relevant, and the recognition of two distinct responses to contact-tracing alerts is astute and important in terms of producing simulations approximating real-world conditions. I greatly appreciate her approach. Additionally, the author’s analysis and description of the results of the study are very clear and complete. I enjoyed and appreciated reading that. However, there are also several areas where I feel the study could be improved, which I detail for the author below.

1. Assumptions of the model:

- Why did the author decide to add “superspreaders” to the model? This may well be a valid decision, but there is no explanation given. An explanation justifying this decision is important, as there are countless other conditions that the author also could have chosen to add to the model but did not. Why this one and not any of the others?
- Along the same lines, why does the author choose not to include the proportion of the population that is vaccinated? Would including it significantly change the differences you found between type-T populations and type-Q populations, and therefore change your conclusions?
- Is mask-wearing and social-distancing accounted for in the model? These are very common behaviors, and including these things would likely affect your results if they are not already accounted for. The contact-tracing app can only alert an individual if they were in proximity to an infected individual, not whether or not they were 5 feet apart vs. 7 feet, nor whether or not either was wearing a mask. A population that adheres to social-distancing and mask-wearing policies would most certainly reduce the proportion of type-T individuals who become infected and spread the virus before they are tested and receive results. Modeling this could significantly reduce the difference in the effects of type-T behaviors vs. type-Q behaviors. Adding this as a variable for comparison in your model might further increase its value in terms of a tool to inform policy decisions around the pandemic.
- The author may also want to include a way to vary presumed infectiousness of the viral variant, since this is an inevitable as long as the pandemic continues. Including this may also significantly change the differences you see between type-T and type-Q populations, and again might make your model more relevant.

2. Public access to your model:

I understand from your statement that the specific data from the Japanese census that you used to populate your model are not available publicly. However, is your model itself publicly accessible for people to populate with their own data? While it is not possible to run simulations using the exact dataset that you used, it would be possible to validate the model itself using a different dataset if it were publicaly available (for example, the Covasim model of Kerr et al.)

3. Related to point 2 above, it would be helpful for the author to make explicit in the paper that the Covasim model by Kerr et al. is in fact open-source. The link to the model should also be included, perhaps in a footnote.
4. Throughout the article, the author refers to the apps “requiring” a particular behavior (testing alone, or quarantine). The apps themselves do not require any particular behavior; they are simply a source of information, and it is the individual who determines their behavior. For this reason, a more precise orientation would be to refer to a “type-T response”, or a “type-Q behavior” as this reviewer has done in the summary section above.
5. I found Section 4.1 on comparison between SIR and ABM very interesting and informative. These are things that I do not have expertise in. However in the context of the author’s article, this discussion seemed out of place. SIR is not mentioned at any other point in the article, and comparison of the relative merits of SIR and ABR is not listed as an objective of the study. I believe that the use of ABR for your study is appropriate and stands on its own in the context of this work, and there is no need to describe or justify why this method was chosen instead of SIR. If you feel it belongs in the paper, I would suggest a short explanation in your conclusions section, or a note in your appendix.

Other suggested improvements:

1. Clarify imprecise or confusing terminology:
   - For example, “superspreader” is not well-defined in its current usage. As the reference the author cites points out (Cave, 2020), “superspreader “is sometimes used to refer to an individual who may shed much more virus than most infected people and who is therefore more infectious, and it is sometimes used to refer to an event in which conditions are such that a large number of individuals become infected (a very crowded event with a long timeframe, for example). It was sometimes unclear what the author was referring to when the term was used. I ultimately determined that the author was referring to an individual, but this needs to be made clear from the start. Additionally, there is one place in the paper [**p. 3, Section 2 (Model), line 4**] where the author specifically uses the term “superspreading environments” which further adds to the confusion.
   - “Lockdown” as it is used in the paper is another confusing term. I believe what the author means by this term is isolation or quarantine of an individual. However the common understanding of the term “lockdown” (in American culture at least) In addition, at one point [**p. 1, line 2-3**] the author uses the term and associates it with not only quarantine but also with behaviors such as social-distancing (in the US this term is specifically used to indicate individuals from different households staying at least 6 feet apart while in public).
2. Please add references in the following locations: p.2 line 2, following “… belong to the former category.”; p. 4, paragraph 2, line 2, after “… asymptomatic”.
3. p. 5, footnote 7: The author notes that in reality it takes 1-3 days to get test results. Why did then did the author choose to use 1 day in your model rather than using the mean (1.5 days)?
4. p. 11, line 2, after “…peak.” Add “(see fig. 3(c)). p. 11 line 3, what is meant by “subsidies”?
5. The author’s point on p. 11 would have more clarity and impact if you specify how many people the 0.3% represents in your model. Further, something like “If x% of the infected are severely ill the means x individuals would require hospitalization. For this model population there would be x hospital(s) with x beds. Thus in this scenario the hospitals would be overwhelmed” In other words, without some clear numbers to go with the author’s statements, they may easily sound like assertions without facts to back them up.
6. p. 12, figure 3, the x-axis does not represent “rates”. Consider labeling the x-axis to make it easier to interpret.
7. p. 19, paragraph 2, lines 10-11: Aren’t “isolation” and “quarantine” referring to the same thing here? If not please clarify.
8. p. 20, final sentence is very unclear. I am not sure what the author is trying to convey.
9. p. 23: Say a bit more about the effects/results under the other 2 conditions.
10. Font size in the figures is quite tiny, and I had difficulty reading it even with magnification. I suggest that the author increase it to 8 point. The author might consider shorter figure titles with a larger font. In some cases part of the figure title can be moved to an axis (see for example Figure 4).
11. The paper would benefit by use of a manuscript editing service, as there are places where wording and language are confusing, unclear, or incorrect.

To conclude, I would like to re-iterate how much I appreciate the topic and approach of this study. I really like the big idea behind it, and feel that it is both relevant and interesting. I thank the author for their very hard work, and I am grateful for the opportunity I was given to review this paper. I do hope that the author finds my comments useful, and also that this paper will be re-submitted following revisions.
